# Monthly E-Portfolio Drop-in Sessions for Core Psychiatry Trainees: A Peer-Led Initiative

**DOI:** 10.1192/bjo.2025.10310

**Published:** 2025-06-20

**Authors:** Aashna Singh, Mohamed Ali, Eleni Fixter

**Affiliations:** Tees, Esk & Wear Valleys NHS Trust, Middlesbrough, United Kingdom

## Abstract

**Aims:** We have observed that many trainees lack confidence in maintaining their e-Portfolio and meeting training requirements. This initiative aimed to provide structured, peer-led support to improve understanding and engagement with portfolio tasks.

**Methods:** We implemented a one-hour, non-mandatory monthly drop-in session, led by core trainees. Topics were selected based on a pre-session survey and further refined through feedback during and after each session. Trainees with experience in specific portfolio components led discussions, with additional guidance from senior (CT3) trainees. Sessions focused on navigating the portfolio, understanding training requirements, and organizing evidence for the Annual Review of Competence Progression (ARCP).

**Results:** Sessions covered topics such as logging clinical assessments, reflective practice, personal development plans, emergency case documentation, and ARCP preparation. Common challenges identified included mapping evidence to competencies, structuring reflections, and creating personal development plans. The peer-led approach facilitated practical, experience-based learning.

**Conclusion:** While online portfolio resources are available, face-to-face peer-led learning provides a valuable supplement in medical education. The monthly drop-in sessions have been well received and have enhanced trainees’ confidence in portfolio management. This model could be beneficial if replicated across other NHS trusts to support core psychiatry trainees nationally.

